# A High-Performance Soy Protein Isolate-Based Nanocomposite Film Modified with Microcrystalline Cellulose and Cu and Zn Nanoclusters

**DOI:** 10.3390/polym9050167

**Published:** 2017-05-06

**Authors:** Kuang Li, Shicun Jin, Hui Chen, Jing He, Jianzhang Li

**Affiliations:** 1 Key Laboratory of Wood Material Science and Utilization, Beijing Forestry University, Beijing 100083, China; kuangli@bjfu.edu.cn (K.L.); jinsc1994@bjfu.edu.cn (S.J.); 2 Ministry of Education, Beijing Key Laboratory of Wood Science and Engineering, College of Materials Science and Technology, Beijing Forestry University, Beijing 100083, China

**Keywords:** soy protein isolate, microcrystalline cellulose, metal nanoclusters, nanocomposite film, tensile strength

## Abstract

Soy protein isolate (SPI)-based materials are abundant, biocompatible, renewable, and biodegradable. In order to improve the tensile strength (TS) of SPI films, we prepared a novel composite film modified with microcrystalline cellulose (MCC) and metal nanoclusters (NCs) in this study. The effects of the modification of MCC on the properties of SPI-Cu NCs and SPI-Zn NCs films were investigated. Attenuated total reflectance-Fourier transformed infrared spectroscopy analyses and X-ray diffraction patterns characterized the strong interactions and reduction of the crystalline structure of the composite films. Scanning electron microscopy (SEM) showed the enhanced cross-linked and entangled structure of modified films. Compared with an untreated SPI film, the tensile strength of the SPI-MCC-Cu and SPI-MCC-Zn films increased from 2.91 to 13.95 and 6.52 MPa, respectively. Moreover, the results also indicated their favorable water resistance with a higher water contact angle. Meanwhile, the composite films exhibited increased initial degradation temperatures, demonstrating their higher thermostability. The results suggested that MCC could effectively improve the performance of SPI-NCs films, which would provide a novel preparation method for environmentally friendly SPI-based films in the applications of packaging materials.

## 1. Introduction

Driven by environmental problems caused by using petroleum-derived polymers, the demand for the development of eco-friendly materials for agriculture, packaging, and coating industries has greatly increased [1]. Due to their renewability and biodegradability, biopolymer materials made from natural resources such as proteins, cellulose, and polysaccharides are regarded as an attractive option in the development of environmentally friendly materials [2]. It is reported that nanoparticles had an effect on protein-based films, but the tensile strength was still relatively poor [3]. In order to further improve the mechanical properties of the protein film, we prepared a novel nanocomposite film modified with microcrystalline cellulose (MCC).

Compared with other proteins, soy protein has been extensively investigated for the development of biodegradable films due to its abundance, low cost, biodegradability, and biocompatibility [4,5]. Soy protein isolate (SPI) is a mixture of proteins containing approximately 90% globulins. 7S and 11S, which amount to about 37% and 31% of major globulins of SPI, have good film-forming ability and polymerization [6,7]. However, relative low strength and poor water resistance have become the main drawbacks of natural SPI-based products and limit further application. Therefore, efforts should be made to modify their properties for commercial application.

In recent years, growing interest has been devoted to materials derived from functional nanomaterials mainly due to its great potential for applications in the fields of biology, biomedicine, energy conversion, catalysis, and chemical sensors [8]. Metal nanoclusters (NCs, <2 nm), emerging as a novel functional nanomaterials, are defined as isolated particles composed of a few to hundreds of atoms [9]. The dimension of this particle approaches the Fermi wavelength of electrons. Their discrete energy levels of electrons make their optical, electronic, and chemical properties markedly different from conventional bulk materials [10]. Due to its reduced size, this particle exhibits even higher surface-to-volume ratios than those of conventional nanoparticles [11]. Most importantly, reports have revealed that metal NCs can have an obvious effect on the physicochemical properties and the subsequent biological responses of natural polymers [12]. Particularly, among other noble metals, Cu NCs and Zn NCs have exhibited significant advantages such as relative high biocompatibility, water solubility, and excellent stability, which makes them attract considerable interest and affords more possibilities for potential applications in various areas [13]. In our previous research, we prepared a SPI-based film modified with Cu NCs and Zn NCs. However, these films exhibited drawbacks such as relatively low mechanical and hydrophobic properties. Therefore, further research on the modification of SPI-NCs films still needs to be done.

As the most abundant biopolymer in nature, cellulose has been widely used in textiles, agriculture, and the food industry [14,15]. Microcrystalline cellulose (MCC) is a cellulose derivative obtained mainly from the acid hydrolysis of wood fiber [16,17]. It is an anionic biopolymer with a relatively high cellulose content and high crystallinity, and the reinforcing phase has a high aspect ratio and has bending strength in synthetic composites [18]. Due to its renewable nature, large specific surface area, and unique physicochemical properties, MCC has great promising applications in biocomposites, protein immobilization, drug delivery, and metallic reaction templates [19]. Recently, the modification of MCC in polymer composites has been a subject of intense research, such as protein, chitosan, wheat bran, and corn starch [20,21,22]. Unfortunately, MCC/SPI composite films still exhibit relatively poor performances in terms of mechanical and permeability properties and need further research.

In this study, in order to further improve the tensile strength, water resistance ability, and thermal stabilities of SPI-based films modified with Cu NCs and Zn NCs, we modified this nanocomposite film with microcrystalline cellulose (MCC). The effects of the modification of MCC on the properties of SPI–NCs films were investigated. These composite films were characterized by Fourier transform infrared spectroscopy (FTIR), X-ray diffraction (XRD), and scanning electron microscopy (SEM). Mechanical properties, water resistance ability, and thermal stabilities were also investigated. The effects of these functional properties might have great importance in the applicability of SPI-based films as renewable and biodegradable packaging materials.

## 2. Materials and Methods

### 2.1. Materials

SPI with 95% protein content was provided by Yuwang Ecological Food Industry Co., Ltd. (Shandong, China). Microcrystalline cellulose [MCC, (C_6_H_10_O_5_)*_n_*] was provided by Sinopharm Chemical Reagent Co., Ltd. (Beijing, China). Copper sulfate anhydrous and zinc chloride purchased from Beijing Chemical Works (Beijing, China) were used to prepare Cu NC and Zn NC solutions. Glycerol with 99% purity and sodium hydroxide of analytical grade were purchased from Beijing Chemical Reagents Co., Ltd. (Beijing, China). All solutions were prepared with deionized water.

### 2.2. Fabrication of SPI-Cu NCs and SPI-Zn NCs

Four grams of SPI was dispersed in 80 mL of distilled water with a magnetic stirrer to prepare SPI solutions. Then, 8 mL of CuSO_4_ or of ZnCl_2_ (20 mmol/L) was dispersed in these SPI solutions. The pH of the mixture was adjusted to about 12 with a NaOH solution and constantly stirred at 25 °C for 10 min. The mixed solution was heated with a magnetic stirrer at 75 °C for 8 h to prepare Cu NCs and Zn NCs.

### 2.3. Preparation of SPI-MCC Nanocomposite Films

The SPI-MCC films were prepared by casting. Two grams of glycerol (50% of SPI, *w*/*w*) and 1.6 g of MCC (40% of SPI, *w*/*w*) were dispersed in the SPI-Cu NC or SPI-Zn NC solutions prepared beforehand. The mixed solutions were heated with a water bath at a temperature of 85 °C for 30 min. Then, the solutions were subsequently poured into leveled Teflon plates and cast with the same amount (40 g). Films were dried in a vacuum drying oven at 45 °C for 24 h and placed in a controlled chamber under the condition of 25 °C and 50% relative humidity for a week before testing.

### 2.4. Characterization of SPI–MCC Nanocomposite Films

#### 2.4.1. High Resolution Transmission Electron Microscopy (HRTEM)

HRTEM images of SPI-Cu NCs and SPI-Zn NCs were performed on FEI Tecnai G2F20 transmission electron microscopy at 200 kV of acceleration voltage (FEI, Hillsboro, OR, USA).

#### 2.4.2. Attenuated Total Reflectance–Fourier Transform Infrared Spectroscopy

Structural characteristics of SPI-based films were investigated with attenuated total reflectance-Fourier transformed infrared (ATR-FTIR) spectra on a spectrometer (Nicolet Nexus 6700, Thermo Scientific, Madison, WI, USA), and the scan width was 4000–650 cm^−1^ through 32 scans.

#### 2.4.3. X-ray Diffraction Analysis

X-ray diffraction (XRD) was carried out to investigate structural changes of the different films on a D8 Advance diffractrometer (Bruker AXS, Karlsruhe, Germany) with a Cu Kα radiation source in continuous scanning mode. The samples were scanned with a range from 5° to 60° and a step interval of 0.02° at a voltage of 45 kV.

#### 2.4.4. Scanning Electron Microscope

Scanning electron microscopy (SEM) (SU8010, Hitachi, Tokyo, Japan) was carried out to characterize the cross-section morphologies of the specimens with an acceleration voltage of 5 kV.

#### 2.4.5. Mechanical Properties

Tensile properties were examined using a tensile testing machine (INSTRON 3365, INSTRON, Norwood, MA, USA) at a speed of 20 mm/min at 25 °C and 50% relative humidity according to report by Li *et al.* [23]. The initial gauge length of each sample was 50 mm. The average values of tensile strength (TS), elongation at break (EB), and Young’s modulus (E) was calculated with five specimens (80 mm × 10 mm^2^).

#### 2.4.6. Thermogravimetric Analysis

The Q50 TGA analyzer (TA Instrument, New Castle, DE, USA) was carried out to investigate the thermal stability of SPI-based films. Each sample was dried at 105 °C for 24 h in an air-circulating oven before testing. Film specimens were scanned from 25 to 600 °C at a heating rate of 10 °C/min under a nitrogen atmosphere (100 mL/min).

#### 2.4.7. Contact Angles Determination

The surface hydrophobicity of each film was measured by water contact angle with a contact angle meter (OCA20, Dataphysics Co., Ltd., Filderstadt, Germany). A film sample (20 × 80 mm^2^) was placed on a movable carrier and leveled horizontally. Each sessile droplet of distilled water was controlled at 3 μL and dropped onto the surface of films. Five replicates were measured for each film.

#### 2.4.8. Water Resistance

Five samples of each film were conducted to determine their moisture content. Film specimens were weighted at room temperature and marked as initial mass (*m*_0_). Then, the samples were dried in an air-circulating oven at a temperature of 105 °C for 24 h and then weighted again (*m*_1_). Moisture content (MC) of each sample was calculated as follows:

MC (%) = (*m*_0_ – *m*_1_)/*m*_0_ × 100 (1)

Afterwards, samples were submerged in a covered bottle with distilled water (30 mL) at 25 °C for 24 h. Then, the samples were dried in an air-circulating oven at a temperature of 105 °C for 24 h and weighted (*m*_2_). Total soluble matter (TSM) was calculated as follows:

TSM (%) = (*m*_1_ – *m*_2_)/*m*_1_×100 (2)

The water absorption of specimens was measured in a desiccator with P_2_O_5_ desiccant at 0% relative humidity for 48 h, and initial weight was recorded as *m*_3_. Then, the specimens were immersed in a covered bottle with 30 mL of distilled water at room temperature for 24 h. The water on the surface of sample was removed and then weighed again (*m*_4_). Water absorption (WA) was calculated as follows:

WA (%) = (*m*_4_ – *m*_3_)/*m*_3_ × 100 (3)

## 3. Results

### 3.1. Characterization of SPI-Based Cu NCs and Zn NCs

**Figure 1:**
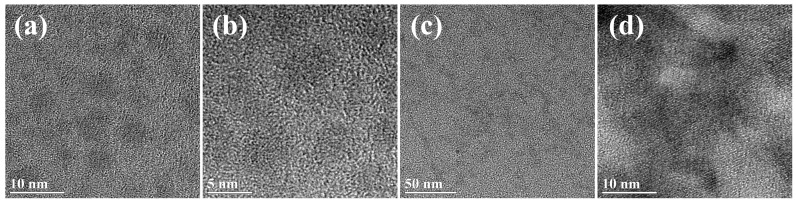
HRTEM images of (**a**,**b**) Cu nanoclusters (NCs) and (**c**,**d**) Zn NCs capped with soy protein isolate.

High-resolution transmission electron microscopy (HRTEM) images were carried out to characterize the morphology of Cu NCs and Zn NCs. Figure 1 demonstrated the presence of Cu NCs and Zn NCs in SPI solutions. Cu NCs were uniform, discrete, and spherical particles with an average diameter about 5 nm (Figure 1a,b). Meanwhile, Zn NCs were also observed in the prepared solution with an average size of less than 10 nm (Figure 1c,d).

### 3.2. Structural Analysis

**Figure 2:**
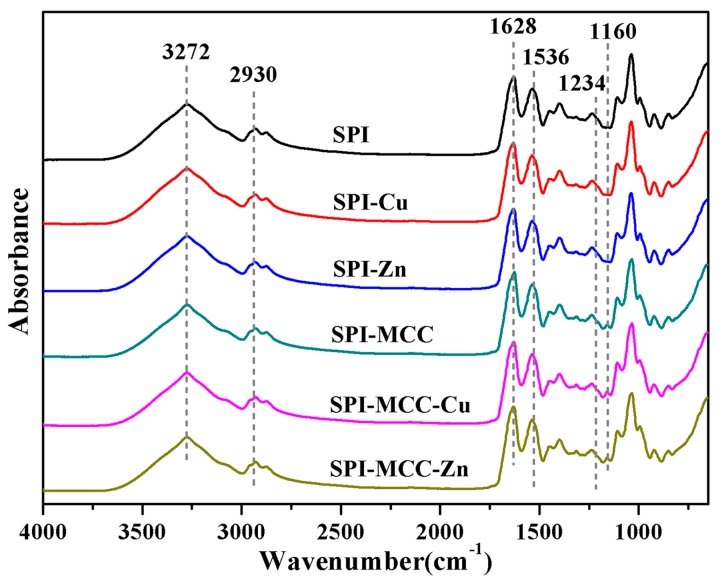
ATR-FTIR spectra of the untreated soy protein isolate (SPI) films and the SPI films modified with Cu NCs, Zn NCs, and MCC.

In order to study the effects of MCC and metal NCs on the structural characteristics of the SPI films, ATR-FTIR spectroscopy analyses (Figure 2) were conducted. The bands at about 3500–3000 cm^−1^ were related to the free and associate O–H and N–H bending vibrations, which formed hydrogen bonding with the carbonyl groups of the peptide linkage of the protein [24]. The absorption bands at 2930 and 2875 cm^−1^ were related to the C–H stretching bands of CH_2_ and CH_3_ groups, and the C–H bending band was observed at 1449 cm^−1^ [25]. The peak at 1038 cm^−1^ belongs to C–O stretching. The characteristic amide bands of soy proteins at 1628, 1536, and 1234 cm^−1^ were assigned to Amide I (C–O stretching), Amide II (N–H bending), and Amide III (C–H and N–H stretching), respectively [23]. These featured bands, which might be affected by hydration and protein-solvent interactions, have been reported [26]. With the addition of MCC, Amides I and II of the SPI–MCC film shifted to 1626 and 1537 cm^−1^, respectively, indicating that the films might expose more polar groups and increase the bindings between the peptide chains [27]. Meanwhile, it was observed that composite films modified with MCC exhibited a new peak at 1160 cm^−1^, which was attributed to the bending mode of C–CH_2_–C, suggesting the unique bands of cellulose [28]. These results demonstrated that molecular hydrogen bonding might be formed between protein molecule chains and polar groups with the modification of MCC, resulting in a cross-linked structure and an improvement in the mechanical properties of the composite films [29].

**Figure 3:**
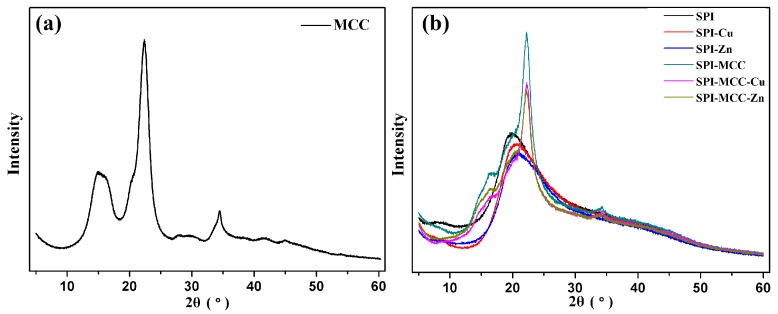
X-ray diffraction patterns: (**a**) MCC powder; (**b**) SPI film and the SPI films modified with Cu NCs, Zn NCs, and MCC.

X-ray diffraction (XRD) patterns of composite films were shown in Figure 3a. The MCC powder exhibited four characteristic crystalline peaks around 2θ = 14.8°, 16.2°, 22.6°, and 34.5°, corresponding to (−1 1 0), (1 1 0), (2 0 0), and (4 0 0) lattice planes, respectively, which was a characteristic of the cellulose I structure [30]. It was reported that the parallel chains in these crystals were strongly inter-molecularly hydrogen bonded [31].

As shown in Figure 3b, the control film showed a strong characteristic peak of SPI at 2θ values of around 20.0°, and SPI-MCC films had strong characteristic peaks of MCC around 2θ = 14.8°, 16.2°, 22.6°, and 34.5°. The sharp peak at 22.6° of the MCC-modified films indicated that the cellulose I structure of MCC had been maintained. The films modified with MCC exhibited relatively high peaks, indicating that MCC had a high crystalline structure and thus increased the rigidity of the films. Compared with SPI-MCC films, the intensity of these peaks of the films modified with MCC and metal NCs were greatly decreased, which was probably caused by the reason that the original crystalline structure of MCC had been partially destroyed during the interactions with metal NCs [32]. The crystalline structure had collapsed and exposed more active groups and prompted more reactions, which resulted in the reduction of the crystalline structure of the modified films [33].

### 3.3. Micromorphology

**Figure 4:**
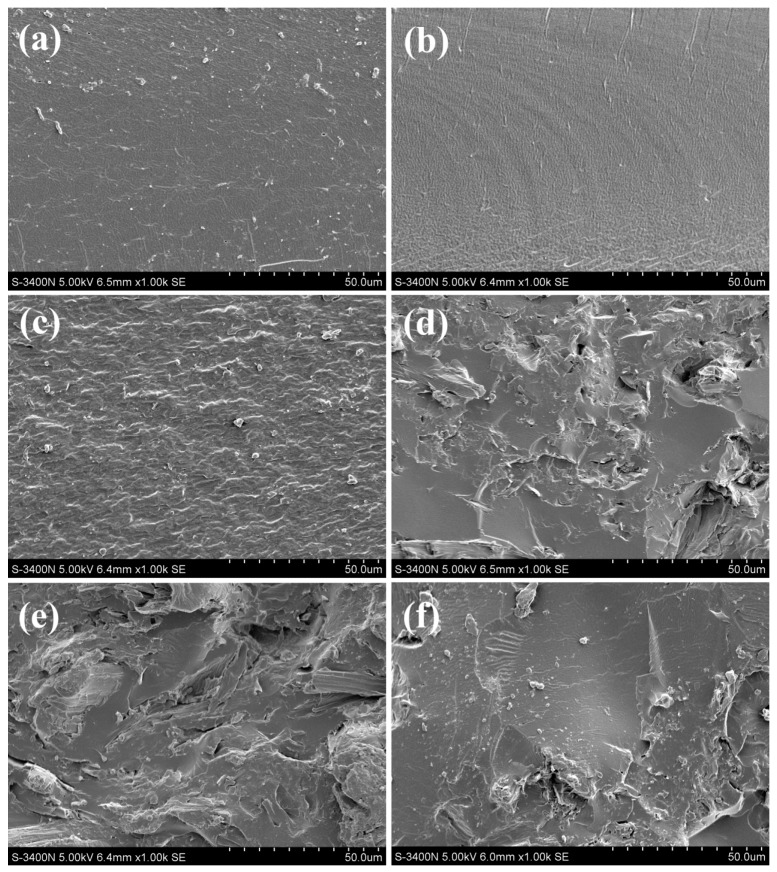
SEM micrographs of the fracture surface of SPI-based films: (**a**) SPI film; (**b**) SPI-Cu film; (**c**) SPI-Zn film; (**d**) SPI-MCC film; (**e**) SPI-MCC-Cu film; (**f**) SPI-MCC-Zn film.

The cross-section morphology of the films was observed by SEM micrographs. As seen in Figure 4a, the SPI film without modification exhibited a relatively smooth and continuous surface, indicating that there were less physical interactions in SPI films. The images (Figure 4b) showed that the cross-sections of SPI-Cu NC film was smoother and more homogeneous, indicating that Cu NCs were well dispersed in the SPI matrix. From Figure 4c, the SPI–Zn film showed a relatively coarse and regular surface, which revealed the good compatibility and uniform distribution of SPI and Zn NCs. However, with the addition of MCC, the films became rather coarse and fluctuant, the presence of cracks and scratches were also observed in the fracture surface (Figure 4d–f), demonstrating the entanglement and strong interactions between SPI and MCC, thereby resulting in a tough fracture and a cross-linked structural formation in the film [34]. These findings confirmed that MCC had a great influence on the structure of the SPI–NC films, thus enhancing the mechanical properties discussed above.

### 3.4. Physical and Mechanical Properties

**Figure 5:**
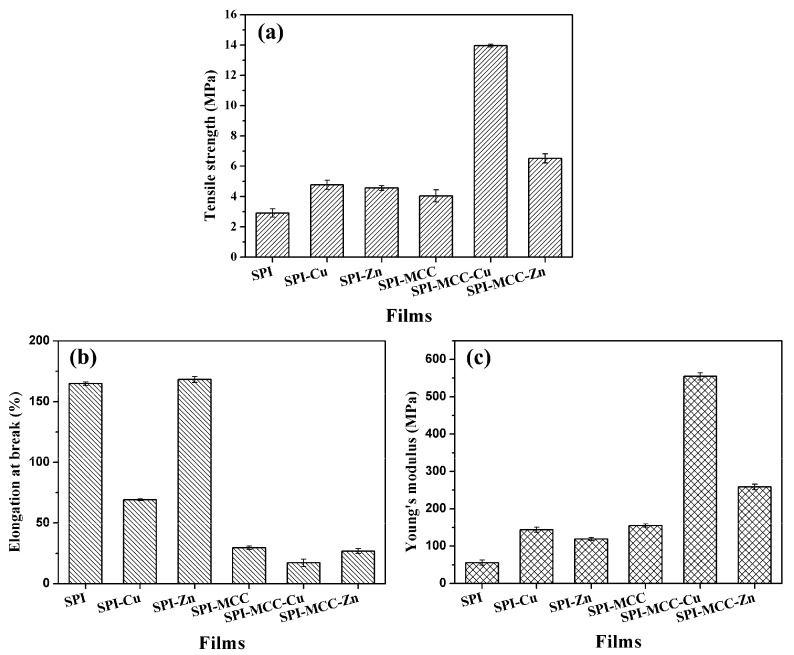
Mechanical properties of SPI-based films: (**a**) tensile strength (TS); (**b**) elongation at break (EB); (**c**) Young’s modulus (E).

**Figure 6:**
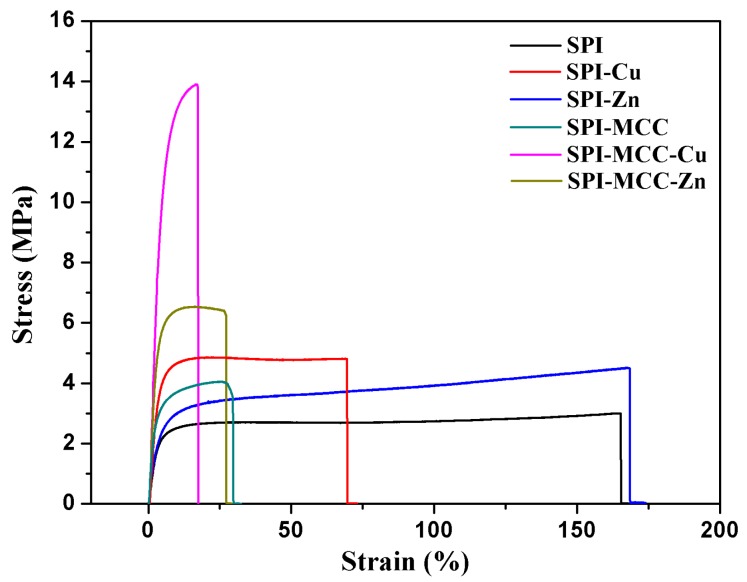
Stress-strain curves of the untreated SPI films and the SPI films modified with Cu NCs, Zn NCs, and MCC.

The mechanical properties of the composite films are shown in Figure 5 and Table 1. Stress–strain curves of films are shown in Figure 6. The stress continuously increased with the increasing strain until the curves were broken without necking, indicating the isotropy of the films. Generally, films formed from purely SPI tend to be brittle, so the SPI film exhibited relative low mechanical properties. The TS values of the SPI-Cu and SPI-Zn films were 4.78 and 4.56 MPa, which was probably due to the increase in the number of potential intermolecular interactions, leading to a higher degree of cross-linking in the nanocomposite films. Compared with the untreated SPI film, the incorporation of MCC also increased the TS from 2.91 to 4.04 MPa, indicating the greater strength of the films. This result was in accordance with those studies reported before [35]. Furthermore, the SPI-MCC-Cu films exhibited the highest TS with the value of 13.95 MPa. The reason was related to the interfacial interaction between the composite matrix and NCs [36]. This fact was probably due to the MCC′s increasing the number of potential intermolecular interactions, which led to a higher degree of cross-linking in the nanocomposite films [37]. In addition to plasticizing the protein matrix, the cellulose microfibrils might also interact more preferentially with the NCs, thereby enhancing the protein-fiber interfaces.

Flexibility is also an important mechanical property of the soy protein film, and this fact was reflected by the EB. The untreated SPI film exhibited a relatively high flexibility with the EB of 164.90%. After being modified with NCs, the SPI-Zn film showed a higher EB than that of the SPI-Cu film, indicating that Zn NCs had a better performance on the flexibility of the films. However, the EB of the SPI films modified with MCC obviously decreased. Since the heterogeneous combination of celluloses and protein might have an important effect on the interfacial stress transfer of composite films, the films modified with MCC all became stiff and exhibited lower flexibility [38].

### 3.5. Thermal Stabilities

**Figure 7:**
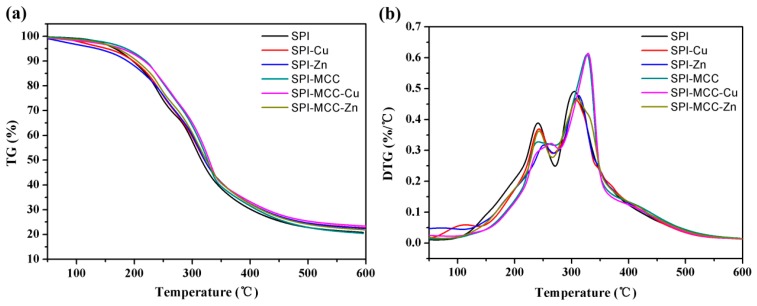
(**a**) Thermo gravimetric (TG) and (**b**) derivative thermo gravimetric (DTG) curves of the untreated SPI films and the SPI films modified with Cu NCs, Zn NCs, and MCC.

The properties of the thermal stabilities of the films was tested by thermal gravimetry (TG) and derivative thermal gravimetry (DTG), and the results are illustrated in Figure 7 and Table 2. Thermal gravimetric curves of the films consisted of three stages (Figure 7a). The initial weight loss from 50 to 120 °C was attributed to the evaporation of residual moisture in the SPI film, while the second stage from 120 to 270 °C was mainly assigned to the evaporation of glycerol, and the third stage from 270 to 450 °C was related to the backbone peptide degradation [39]. Particularly, the main weight loss stage at 350 °C was caused by the degradation of carbonaceous matter in MCC [40].

As listed in Table 2, the initial degradation temperatures (*T_i_*_2_) of the SPI films modified with MCC were higher than that of other films, indicating a presence of abundant hydrogen bond groups formed in the protein chains [41]. Moreover, the SPI-MCC-Cu film had the highest degradation rate (*T*_max2_) at the highest temperature in the DTG curve compared with the control film, markedly increasing from 304.85 to 329.06 °C, which might be attributed to the cross-linking reactions among MCC, NCs, and SPI. Therefore, we can conclude that the thermal stability of the SPI-NC films was improved by the modification of MCC.

### 3.6. Water Resistance

**Figure 8:**
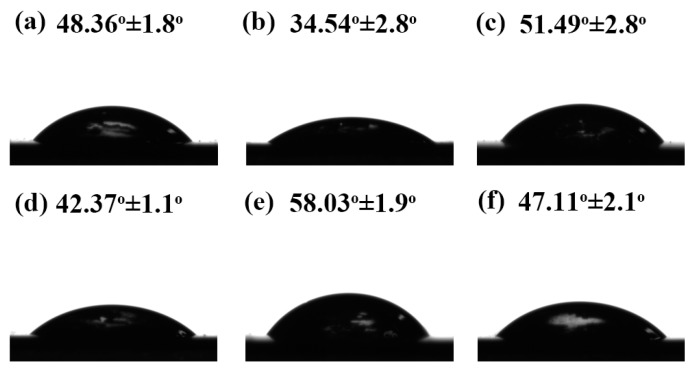
Water contact angles of SPI-based films: (**a**) SPI film; (**b**) SPI-Cu film; (**c**) SPI-Zn film; (**d**) SPI-MCC film; (**e**) SPI-MCC-Cu film; (**f**) SPI-MCC-Zn film.

Measuring the contact angle of the water droplets on the surfaces was an important step in investigating the hydrophobicity of film surfaces. Generally, a high contact angle indicated the high hydrophobicity of the surface. As shown in Figure 8 and Table 3, the control film presented a contact angle of about 48.36°, corresponding to a relatively high hydrophilic surface. After being modified with NCs, the SPI-Cu and SPI-Zn films exhibited contact angles of 34.54° and 51.49°, indicating that Zn NCs demonstrated a superior performance on the surface hydrophobicity of the SPI film. With the addition of MCC, the water contact angle of the SPI-MCC film reduced to 42.37°, indicating a decrease in the hydrophobicity of the films. Similar observations have been previously reported for MCC films [42]. However, it was worth noting that the SPI-MCC-Cu film represented a marked increase in the contact angle with a value of 58.03°, which was highest among all films. This result suggested that this composite film might expose more polar groups and hydrophobic amino acids to the surface, thus improving the water resistance [43].

The water resistance of the SPI-based films could be characterized by moisture content (MC), which was considered as an important property from a packaging materials perspective. The amount of water present in the films was determined by MC values, which are shown in Table 3. The MC of the unmodified SPI film was relatively high, and there was no obviously difference between the SPI films and the films modified with metal NCs, indicating that the addition of NCs did not significantly affect the water resistance of the SPI films. However, the MC of the SPI film decreased from 15.80% to 13.40% when MCC was incorporated, and the SPI-MCC-Cu film exhibited the lowest MC value—11.68%. This could be justified by the fact that MCC and metal NCs brought significant barrier properties and there were some specific interactions among the MCC, NCs, and SPI that stabilized the film structure [44], which is consistent with the ATR-FTIR and XRD results.

Total soluble matter (TSM) and water absorption (WA) were also considered as the indicators of the films’ resistance to water. Generally, lower values would indicate superior water resistance. Compared to the control film, the TSM and WA values of the films modified with NCs were slightly greater. With the addition of MCC, the values of both TSM and WA significantly decreased from 36.79% and 197% to 29.63% and 119%, respectively. Furthermore, the TSM and WA values of the SPI-MCC-Cu film were the lowest among the other films, decreasing to 94.86% and 10.13%, indicating there was a remarkable improvement in water resistance of the composite film after modification. The results confirmed that the molecules of MCC and NCs might greatly crosslink the polymeric network of SPI and decrease the swelling of the soy protein matrix under high moisture [45,46]. Therefore, the SPI-MCC-Cu films prevented the permeation of water molecules and exhibited lower TSM and WA values.

## 4. Conclusions

This study showed the preparation of plant-derived biodegradable materials through the casting methodology of SPI modified with MCC and NCs. FTIR showed the hydrogen bonding and cross-linking interaction in the film. The efficiency of the crystalline structures of the SPI films changed by the MCC and NCs were evidenced by the XRD patterns. The SPI-MCC-Cu film had the highest tensile strength, increasing from 2.91 to 13.95 MPa. The enhanced entanglement and cross-linking in the microstructure of the films were observed with SEM. The composite films modified with MCC exhibited a higher contact angle and lower MC, TSM, and WA values, which indicated their superior water resistance. Compared to the unmodified SPI film, these composite films also exhibited superior thermal stability. Among these tests, the mechanical properties of the nanocomposite films modified with MCC improved most markedly. Therefore, the use of these protein-based biodegradable materials with improved properties might provide a valuable opportunity to replace conventional petroleum-derived plastics whereby this vast agricultural resource can be employed as a packing material.

## Figures and Tables

**Table 1:** Thickness, tensile strength (TS), elongation at break (EB), and Young′s modulus (E) of the untreated SPI films and the SPI films modified with Cu NCs, Zn NCs, and MCC.

Films	Thickness (mm)	TS (MPa)	EB (%)	E (MPa)
SPI	0.158 (0.023) ^a^	2.91 (0.27)	164.90 (0.07)	55.48 (3.62)
SPI-Cu	0.271 (0.015)	4.78 (0.30)	69.13 (0.03)	144.00 (3.35)
SPI-Zn	0.260 (0.017)	4.56 (0.16)	168.30 (0.12)	118.50 (2.14)
SPI-MCC	0.255 (0.027)	4.04 (0.40)	29.57 (0.07)	154.90 (2.31)
SPI-MCC-Cu	0.335 (0.019)	13.95 (0.09)	17.12 (0.15)	554.70 (4.64)
SPI-MCC-Zn	0.248 (0.020)	6.52 (0.30)	26.82 (0.10)	258.40 (3.73)

^a^ mean (standard deviation).

**Table 2:** Thermo-degradation data of the untreated SPI films and the SPI films modified with Cu NCs, Zn NCs, and MCC.

Films	*T_i_*_1_ (°C)	*T*_max1_ (°C)	*T_i_*_2_ (°C)	*T*_max2_ (°C)
SPI	146.72	239.98	286.55	304.85
SPI-Cu	155.83	241.63	291.94	308.17
SPI-Zn	178.68	251.38	295.28	312.84
SPI-MCC	168.42	235.85	304.94	328.39
SPI-MCC-Cu	183.59	243.26	309.54	329.06
SPI-MCC-Zn	170.44	243.28	305.16	310.81

*T_i_*: initial temperature of degradation; *T*_max_: temperature at maximum degradation rate.

**Table 3:** Water contact angles, moisture content (MC), total soluble matter (TSM), and water absorption (WA) of the untreated SPI films and the SPI films modified with Cu NCs, Zn NCs, and MCC.

Films	Contact Angles (°)	MC (%)	TSM (%)	WA (%)
SPI	48.36 (1.8) ^a^	15.80 (1.2)	36.79 (1.0)	197.32 (8.3)
SPI-Cu	34.54 (2.8)	15.44 (1.8)	38.99 (0.9)	210.21 (6.9)
SPI-Zn	51.49 (2.8)	15.49 (1.5)	36.91 (0.8)	200.06 (6.7)
SPI-MCC	42.37 (1.1)	13.40 (1.7)	29.63 (1.2)	119.86 (7.0)
SPI-MCC-Cu	58.03 (1.9)	11.68 (2.1)	10.13 (0.8)	94.86 (6.5)
SPI-MCC-Zn	47.11 (2.1)	13.96 (1.8)	27.36 (1.0)	162.88 (5.0)

^a^ mean (standard deviation).
